# Cardiac Rehabilitation: Far Beyond Coronary Artery Disease

**DOI:** 10.5935/abc.20160002

**Published:** 2015-12

**Authors:** Claudio Gil Soares de Araújo

**Affiliations:** Instituto do Coração Edson Saad - Universidade Federal do Rio de Janeiro - Rio de Janeiro, RJ; Clínica de Medicina do Exercício - CLINIMEX - Rio de Janeiro, RJ - Brazil

**Keywords:** Cardiovascular Diseases, Physical Exercise, Exercise Training, Exercise Programs, Flexibility

Cardiac rehabilitation (CR), as originally proposed in the mid of last century, was primarily aimed at returning life back to normal, with emphasis on aspects of physical, mental and professional plenitude. At that time, a few paradigms were modified. Instead of four to six weeks of bed rest and immobilization following acute myocardial infarction, patients were oriented to sit on chairs, which was described as "*armchair treatment*"; right after, studies began to show the favorable results of early ambulation.^1^ This has evolved and currently the Brazilian Society of Cardiology guidelines proposing and advising how to implement and to start supervised exercise programs for CR.^2^ Those guidelines comprise aerobic, muscle strengthening and flexibility exercises to be started as soon as clinical stability is reached after myocardial infarction. Currently CR is aimed at providing patients with better or much better conditions than those prior to the cardiac event or the diagnosis of cardiovascular disease.

Although CR is a frequent and recurring theme of several presentations in scientific meetings of the specialty, being thus known by clinical cardiologists, there is worldwide consensus that only a very small part of patients with coronary artery disease is usually referred by their physicians for CR programs. Several are the reasons for that insufficient referral and they will not be addressed in this editorial.

The present editorial is aimed at alerting to another aspect, certainly less known to clinical cardiologists. CR and especially supervised exercise programs should be recommended not only to patients with coronary artery disease, but also to a wide range of cardiovascular disorders and, thus, to almost all patients with heart disease.

As highlighted in an article recently published in the *Arquivos Brasileiros de Cardiologia*,^3^ aerobic fitness has long been known to be one of the best, if not the best marker of the health status and prognosis of not only healthy individuals, but also those with heart disease. Aerobic fitness is expressed as the maximal oxygen uptake, preferentially measured through expired gas analysis during a cardiopulmonary exercise testing. Although the aerobic fitness is highly influenced by genetics, there is consensus that optimal levels can only be achieved via regular physical exercise, predominantly aerobics. Thus, for both primary and secondary prevention of cardiovascular diseases, being physically active is fundamental to optimize the aerobic fitness. Nevertheless, that is seen neither in daily clinical practice nor in the epidemiological data of the Brazilian population, and most patients with heart disease remain sedentary or insufficiently physically active.

Based on the increasing knowledge on physiological responses and adaptations to physical exercise of individuals from different populations and with different clinical characteristics, several researchers and CR services have recently explored other possibilities of indications and care for 'non-coronary heart patients'. Thus, the risks and benefits of interventions with regular physical exercise can be better known in a wide range of 'non-coronary heart patients', some considered as having absolute contraindications until recently. Those patients can be then referred to CR programs.

The Chart 1 lists 22 cardiovascular disorders,^4-25^ except for coronary artery disease, on which there are studies and scientific evidence suggesting the lack of harm or of important risk and presence of significant benefit resulting from the participation in CR programs and regular physical exercise sessions with or without medical supervision.

**Chart 1:** List of 22 non-coronary cardiovascular conditions that have been shown to clinically benefit from physical exercise intervention

**Number**	**Cardiovascular condition**	**Reference**	**PubMed URL**
1	Heart failure (all functional classes)	(4)	http://www.ncbi.nlm.nih.gov/pubmed/24771460
2	Heart failure with preserved ejection fraction	(5)	http://www.ncbi.nlm.nih.gov/pubmed/25399909
3	Dilated cardiomyopathy	(6)	http://www.ncbi.nlm.nih.gov/pubmed/22739638
4	Heart transplantation (post-surgery)	(7)	http://www.ncbi.nlm.nih.gov/pubmed/25840503
5	Heart valve surgery (post-event)	(8)	http://www.ncbi.nlm.nih.gov/pubmed/22024327
6	Peripheral artery disease	(9)	http://www.ncbi.nlm.nih.gov/pubmed/25766947
7	Systemic arterial hypertension	(10)	http://www.ncbi.nlm.nih.gov/pubmed/23525435
8	Stroke (post-event)	(11)	http://www.ncbi.nlm.nih.gov/pubmed/24142492
9	Transient ischemic attack (post-event)	(12)	http://www.ncbi.nlm.nih.gov/pubmed/25023152
10	Pulmonary arterial hypertension	(13)	http://www.ncbi.nlm.nih.gov/pubmed/26231884
11	Chagas disease	(14)	http://www.ncbi.nlm.nih.gov/pubmed/25098373
12	Congenital heart diseases	(15)	http://www.ncbi.nlm.nih.gov/pubmed/23746621
13	Catecholaminergic polymorphic ventricular tachycardia	(16)	http://www.ncbi.nlm.nih.gov/pubmed/24837260
14	Erectile dysfunction (vascular etiology)	(17)	http://www.ncbi.nlm.nih.gov/pubmed/22435000
15	Neurocardiogenic syncope	(18)	http://www.ncbi.nlm.nih.gov/pubmed/24714795
16	Postural orthostatic tachycardia syndrome	(19)	http://www.ncbi.nlm.nih.gov/pubmed/25487551
17	Abdominal aortic aneurysm (< 55 mm)	(20)	http://www.ncbi.nlm.nih.gov/pubmed/23793234
18	Asymmetric hypertrophic cardiomyopathy	(21)	http://www.ncbi.nlm.nih.gov/pubmed/23928567
19	Left ventricular assistance device (post-implantation)	(22)	http://www.ncbi.nlm.nih.gov/pubmed/25381335
20	Transposition of the great vessels (adults, post-surgery)	(23)	http://www.ncbi.nlm.nih.gov/pubmed/25809342
21	Atrial fibrillation (post-ablation)	(24)	http://www.ncbi.nlm.nih.gov/pubmed/26113406
22	Transcatheter aortic valve implantation	(25)	http://www.ncbi.nlm.nih.gov/pubmed/25593575

The disorders are listed in a decreasing order according to evidence level, level of knowledge available and clinical experience regarding the benefits of regular physical exercise. Each indication is based on a qualified and recent scientific reference, whose abstract in PubMed can be obtained via the hyperlink provided.

In fact, this list should actually be understood as open and not restrictive, because, except for arrhythmogenic right ventricular dysplasia,^26,27^ end-stage heart failure and acute myocardial or heart valve infections, some benefit can almost always be obtained from participating in CR programs or supervised physical exercise sessions, as long as the patient with heart disease is stable from the clinical, hemodynamic and electrocardiographic viewpoint.

I hope this editorial helps clinical cardiologists to definitively incorporate, as major priority, regular physical exercise into the treatment of patients with heart disease, not only the already known 'coronary heart patient', but also and with great emphasis on the 'non-coronary heart patient'. In parallel, public and private hospitals and clinics of Cardiology and Exercise Medicine should prepare to properly approach this new demand.
